# A New Rapid Method for the Authentication of Common Octopus (*Octopus vulgaris*) in Seafood Products Using Recombinase Polymerase Amplification (RPA) and Lateral Flow Assay (LFA)

**DOI:** 10.3390/foods10081825

**Published:** 2021-08-06

**Authors:** Amaya Velasco, Graciela Ramilo-Fernández, Françoise Denis, Luís Oliveira, Peter Shum, Helena Silva, Carmen G. Sotelo

**Affiliations:** 1 Instituto de Investigaciones Marinas (IIM-CSIC), Eduardo Cabello 6, 36208 Pontevedra, Spain; graciela@iim.csic.es (G.R.-F.); carmen@iim.csic.es (C.G.S.); 2 BOREA MNHN, CNRS 8067, SU, IRD 207, UCN, UA-BIOSSE Le Mans Université, 72000 Le Mans, France; francoise.denis@univ-lemans.fr; 3 Instituto Português do Mar e da Atmosfera (IPMA, I.P.), Av. Dr. Alfredo Magalhães Ramalho 6, 1495-165 Algés, Portugal; luis.oliveira@ipma.pt (L.O.); hsilva@ipma.pt (H.S.); 4 School of Biological and Environmental Sciences, Liverpool John Moores University (LJMU), Liverpool L2 2QP, UK; p.shum@ljmu.ac.uk

**Keywords:** Octopus, *Octopus vulgaris*, RPA, Lateral Flow detection, species authentication, seafood control

## Abstract

The common octopus (*Octopus vulgaris*) is a highly valued cephalopod species which is marketed with different grades of processing, such as frozen, cooked or even canned, and is likely to be mislabeled. Some molecular methods have been developed for the authentication of these products, but they are either labor-intensive and/or require specialized equipment and personnel. This work describes a newly designed rapid, sensitive and easy-to-use method for the detection of *Octopus vulgaris* in food products, based on Recombinase Polymerase Amplification (RPA) and a detection using a Lateral Flow assay (LFA). After studying several gene markers, a system of primers and nfo-probe was designed in the COI (Cytochrome Oxidase I) region and was successfully tested in 32 reference samples (covering 14 species) and 32 commercial products, after optimization. The method was also validated in a ring trial with eight European laboratories and represents a useful tool for food authenticity control at all levels of the value chain.

## 1. Introduction

Food fraud, while not a new phenomenon, has become more relevant in recent years, and seafood is one at the highest risk categories of foods [1]. Seafood fraud practices (including intentional mislabeling and species substitution) can take place at multiple points along the value chain, where fraudsters benefit from the fact that most consumers have difficulties to visually identify the product at species levels [2].

Current methods for species authentication involve the use of molecular approaches, such as proteins or DNA markers, that can be analyzed using simple or more sophisticated, time-consuming or rapid, costly or affordable methods [3]. The overall objective of authentication methods should be their reliability and easiness of implementation, while preserving specificity, sensitivity, and robustness [4].

Seafood authentication is mostly performed nowadays using DNA analysis techniques, and most control laboratories rely in PCR (Polymerase Chain Reaction)-based methods, such as FINS (Forensically Informative Nucleotide Sequencing) or specific qPCR methods [5]. Authentication methods should account for the fact that seafood can be marketed with very different levels of processing, ranging from fresh and frozen, in which biomolecules are almost intact, up to extensively thermally processed, such as the case of canning, where even DNA can be damaged and only short DNA fragments of about 200 bp (base pair) or less are present in these products [6].

Authentication methods are needed in different situations: they can be used for regulatory enforcement by control laboratories, for the control of ingredients sourcing by dedicated laboratories or by staff not specialized in the industry or field situation (i.e., border controls). In some of these cases, it is not always possible to have molecular biology equipment available [7] and, therefore, rapid and easy-to-use methods are needed, at least for screening purposes.

Cephalopods represent an important part of the global fisheries, and involve hundreds of species, some of them with a high market value [1]. One of the most valued cephalopods is the Common Octopus (*Octopus vulgaris*), a benthic worldwide distributed species, which is captured both by artisanal and industrial fisheries. The world catches for this species in recent years range from 35,000 to 40,000 tons [8]. This species is especially relevant for Southern European countries such as Portugal, Spain, Italy and Greece. Prices reached record levels during 2018 as a reflection of a growing demand and a decline of the landings, mainly due to the more restrictive regulations in producing countries, such as Morocco, Mauritania and Spain, to increase the protection of the resource [9]. Additionally, it presents a wide range of market presentations, making it susceptible to species substitution, and in fact, it is one of the most commonly mislabeled species [10], with levels of mislabeling higher than 50% in some countries [11].

For the authentication of cephalopod species, different molecular methods, such as Forensically Informative Nucleotide Sequencing (FINS) and barcoding, have shown effective for identification [12] and have been widely used for control purposes. Additionally, some other PCR-based methods for the detection of mislabeling in cephalopods, including *O. vulgaris (Octopus vulgaris)*, have been developed, such as RAPD (Random Amplified Polymorphic DNA) [13], PCR-RFLP (Restriction Fragment Length Polymorphism) [14] and Real time-PCR [15]. Nevertheless, all these methods have drawbacks, such as technical complexity and/or being time-consuming, and they are also based on the PCR technique, which requires precise temperature control and rapid thermocycling steps, which implies a high optimization effort and expensive equipment.

Recombinase Polymerase Amplification (RPA) is a novel and isothermal alternative to PCR, since it does not require thermal denaturation of the template, such as PCR, and operates at a low and constant temperature [16]. RPA is also remarkable due to its simplicity, high sensitivity, selectivity, compatibility with multiplexing and rapid amplification [17]. Since its appearance in research in 2006, this approach in combination with diverse detection methods has been used for diagnostic applications [18,19], and less frequently for food analysis [20,21,22]; however, to the authors’ knowledge, it has not yet been used in the field of seafood authentication, where it is very important to have rapid and portable methods to authenticate both raw materials and marketed products [7].

Lateral Flow strips are used to visualize results from specific DNA amplifications, a technique that adds the advantages of rapidity and one-step analysis without any equipment, a low operational cost, a user-friendly format with visual results, high specificity and sensitivity and portability [23]. Lateral flow analysis in combination with RPA (RPA-LFA) constitutes a “point of care testing” type and widely affordable nucleic acid-based test.

The purpose of the work is to develop a rapid method for the authentication of the common octopus (*Octopus vulgaris*) in seafood products using Recombinase Polymerase Amplification (RPA) and Lateral Flow Assay (LFA), providing a rapid and effective tool for the authenticity control of products containing *O. vulgaris*, with a high potential of commercialization, as it can be easily implemented at the point of sale or any control point. This work includes an extensive sampling and analysis effort, as well as an interlaboratory validation, which give robustness to the designed method and proves its applicability on the field.

## 2. Materials and Methods

### 2.1. Schematic Overview of the Experimental Program

**Figure 1:**
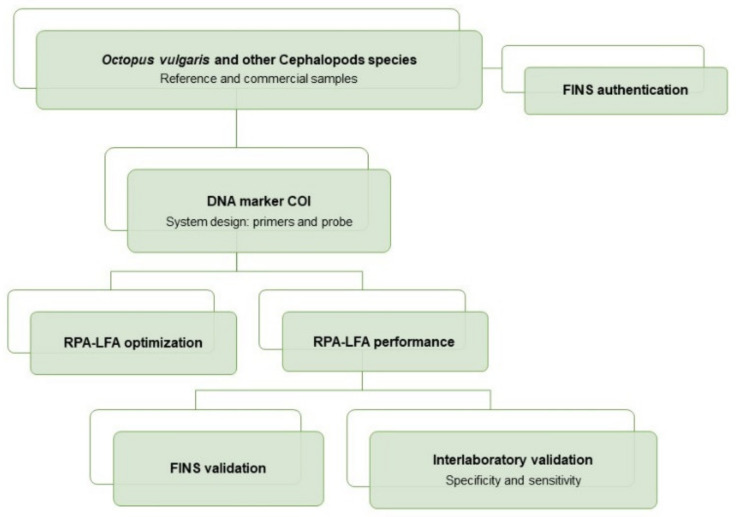
Flow diagram of the experimental procedures used for the development of the RPA-LFA method for the specific detection of *Octopus vulgaris* in seafood products.

An overview of the workflow of this study can be summarized as follows: the sampling included reference and commercial samples of *O. vulgaris* and related species, with all samples being authenticated by FINS (Forensically Informative Nucleotide Sequencing). The RPA system (primers and probe) was designed in a COI mitochondrial DNA region, and the RPA-LFA method was optimized and tested with reference samples and validated with commercial samples. The method validation was performed by comparison of the results obtained with RPA-LFA with the FINS results. Finally, the overall sensitivity and specificity of the method was determined with an interlaboratory test (Figure 1).

### 2.2. Sampling

A total of 15 samples of *O. vulgaris* from Spain, Portugal and Morocco and 17 samples of related species and possible substitutes were used as reference, covering a total of 14 species of 10 genera (Table 1). Furthermore, 32 commercial food products of *O. vulgaris* and other cephalopod species were collected in Spanish supermarkets and restaurants during 2019 and 2020, including fresh, frozen, defrosted, cooked and canned products, for the validation of the method (Table 2). All products were photographed, and the information of the label was registered prior to their storage at −20 °C.

### 2.3. Sample Preparation and DNA Extraction

Prior to the digestion of the tissue, cooked and canned samples were washed in sterile water. DNA was obtained from a portion of 0.3 g of muscle tissue, which was digested at 56 °C in a thermo shaker with 860 µL of lysis buffer (1% SDS (Sodium dodecyl sulfate), 150 mM NaCl, 2 mM EDTA (Ethylenediaminetetraacetic acid) and 10 mM Tris-HCl at pH 8), 100 µL of 5 M guanidinium isothiocyanate and 40 µL of proteinase K (20 mg/mL). After 3 h, 40 µL of extra proteinase K was added and left overnight. DNA was isolated from the digested tissue with the Wizard DNA Clean-up System kit (Promega) following the manufacturer’s protocol. The quantification of the double stranded DNA obtained was performed with the Qubit dsDNA BR Assay Kit (Life Technologies, Carlsbad, CA, USA,) and Qubit 3.0 fluorometer (Invitrogen, Waltham, MA, USA). Purified DNA was stored at −20 °C until further analysis.

### 2.4. Authentication of Samples and Method Validation by FINS

All samples used in this study, both reference and commercial, were identified by FINS before they were tested with the newly developed method. For that purpose, PCR reactions were carried out in a Veriti Thermal cycler (Applied Biosystems, Foster City, CA, USA) in a final volume of 25 µL with Illustra PuReTaq Ready-To-Go PCR Beads (GE Healthcare, Chalfont St Giles, UK), 1 µL of each primer (10 µM) and 100 ng of template DNA. Table 3 shows the sequences of the primers used for PCR and sequencing reactions. Primers designed by Folmer [24] were used to amplify a 750 bp fragment of the mitochondrial COI region with the following thermal protocol: a preheating step of 3 min at 95 °C, followed by 35 cycles of 1 min at 95 °C, 1 min at 40 °C and 1.5 min at 72 °C, with a final extension step at 72 °C for 7 min. For highly processed samples, the 16SVAR primers described by Chapela [12] were used to amplify a 210 bp fragment of the mitochondrial 16S rDNA, with a preheating step of 3 min at 95 °C, followed by 35 cycles of 40 s at 94 °C, 40 s at 50 °C and 40 s at 72 °C, and a final extension step at 72 °C for 7 min. Negative controls were included in all PCR sets.

PCR products were purified with Illustra ExoProStar (GE Healthcare, Chalfont St Giles, UK) and sequencing reactions were performed with BigDye Terminator 1.1 (Applied Biosystems, Foster City, CA, USA), following the manufacturer’s protocol. The automatic sequencing of both strands was carried out in an ABI PRISM 3130 (Applied Biosystems, Foster City, CA, USA). Forward and reverse sequences were analyzed using Chromas and Bioedit [25] and aligned with references from the NCBI (National Center for Biotechnology Information)and the IIM-CSIC (Instituto de Investigaciones Marinas) sequence database. Species identification was performed with the Tamura-Nei distance model and Neighbor-joining phylogenetic tree (1000 bootstrap replicates) using MEGA [26]. The sequences obtained from the samples were also authenticated with BLAST [27], a tool that was also used to check the quality and the coverage of the resulting sequences.

All COI sequences obtained in this study were uploaded to the GenBank [28] of the NCBI (accession numbers are shown in Table 2; Table 3).

### 2.5. Design of Primers and Probe for RPA

**Figure 2:**
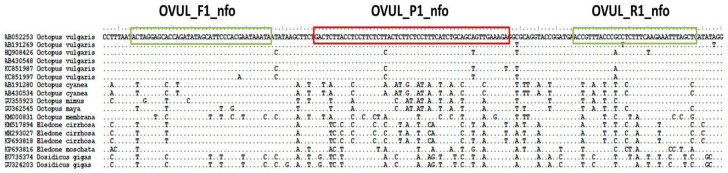
Location of the RPA primers and probe designed in the *Octopus vulgaris* COI region.

Sequences of different mitochondrial and nuclear markers belonging to *O. vulgaris* and related cephalopod species were downloaded from GenBank and aligned using Bioedit. These alignments were analyzed in order to find a suitable fragment for the detection of *O. vulgaris*. The primers (OVUL_F1_nfo, forward, and OVUL_R1_nfo, reverse) and the nfo probe (OVUL_P1_nfo) were designed following the recommended steps of TwistDx Assay design manual (TwistDx, Cambridge, UK), regarding length, G+C content and annealing temperatures. The designed system amplifies a 155 bp fragment of the COI region and entails two primers and a FAM-labeled nfo-probe (Figure 2), of which the sequences are the following:

OVUL_F1_nfo: 5′-ACTAGGAGCACCAGATATAGCATTCCCACGAATA-3′
OVUL_R1_nfo: Biotin-5′-GAGCTAAATTTCTTGAAAGAGGCGGGTAAACGGT-3′
OVUL_ P1_nfo: FAM-5′-ACTCTTACCTCCTTCTCTTACTCTTCTCCTTT[THF]ATCTGCAGCAGTTGA-3′

The reverse primer is modified with a Biotin in 5′, and the probe has a 6-FAM modification in 5′ and a blocking group in 3′ (Spacer C3). THF corresponds to the position of the tetrahydrofuran residue.

### 2.6. RPA-LFA Optimization

RPA reactions were carried out with the TwistAmp nfo kit (TwisDX, Cambridge, UK). RPA conditions were previously adjusted on the basis of the preliminary test carried out with target and non-target species, testing different reagent concentrations (data not shown) and different incubation temperatures (from 25 to 50 °C).

The final RPA-LFA protocol was determined based on the quality of the signal, the manufacturer’s recommendations and the specificity of the test. Each RPA reaction was performed in a total volume of 50 µL, with 1.8 µL of each primer (10 µM), 0.6 µL of the nfo probe (10 µM), 29.5 µL of the rehydration buffer, 2.2 μL of 280 mM magnesium acetate (MgOAc) and 1 µL of DNA template (50 ng/µL). Then, reactions were incubated at 40 °C in a Veriti thermal cycler (Applied Biosystems, Foster City, CA, USA) for 15 min and RPA products were then diluted 1:50 with PBST running buffer. Afterward, 10 μL of the diluted sample was transferred to the sample pad of the Hybridetect strip (Milenia Biotec GmbH, Gießen, Germany) and the strip was placed vertically with the sample pad submerged in 150 μL of PBST running buffer. After 4 min, the result was photographed and registered. The presence of two clear and distinguishable bands (control band and test band) on the strip indicated a positive result, while a negative result showed only the control band. In the cases where the test band was very faint in the established time or not clearly visible in the photograph, the result was considered negative.

### 2.7. Evaluation of the RPA-LFA Performance

#### 2.7.1. Detection Limit

The described method was used for a detection limit assay to determine the lowest quantity of template DNA that can be visually detected by the method. Six serial dilutions of DNA obtained from a reference sample of *O. vulgaris*, from 50 ng (total DNA per reaction) to 50 × 10^−5^ ng, were tested, and a negative control with no DNA was included. The resulting RPA products were visualized both in LF strips as described before, and in a 2% agarose gel.

#### 2.7.2. Specificity, Sensitivity and Application to Commercial Products

All 32 reference samples of *O. vulgaris* and related species were analyzed for the evaluation of the specificity and sensibility of the method. Additionally, 32 commercial samples described in Table 2 were tested for the internal validation with the same procedure.

#### 2.7.3. Interlaboratory Validation

For the validation of the method, an SOP (Standard Operating Procedure) was elaborated and tested in a ring trial with eight participant laboratories from five different European countries (see Supplementary Materials). Ten tissue samples (eight blind samples, one positive and one negative), selected from the used reference samples, were distributed to each laboratory, together with the SOP. The blind samples included *O. vulgaris* and other cephalopod species highly related and/or possible market substitutes (Table 4). The results obtained in this ring test were used for obtaining the sensitivity and specificity of the method, being calculated as follows:

(1) Specificity = [True Negatives/(True Negatives + False positives)] × 100

(2) Sensitivity = [True Positives/(True Positives + False negatives)] × 100

## 3. Results

### 3.1. Design of Primers and Probe

All genetic markers available on GenBank for *O. vulgaris* and related species were analyzed in order to find the best region for the design of the RPA system. This region was found in a short fragment of the mitochondrial COI marker, highly conserved in *O. vulgaris* and with enough nucleotide differences with the rest of the species to design a specific primers-probe system. In particular, the designed probe showed from 11 to 17 nucleotide differences with non-target species. Although RPA can amplify fragments up to 1.5 kb (Kilobase pairs) [18], the optimal length recommended by the manufacturer for a nfo system is 100 to 200 bp, and the fragment amplified by the designed system (155 bp) complies with this recommendation. The objective of amplifying a short fragment was to obtain the highest performance and also to get successful amplifications in cases of highly processed tissues (e.g., canned products), where DNA might be fragmented.

### 3.2. Optimization of RPA-LFA

**Figure 3:**
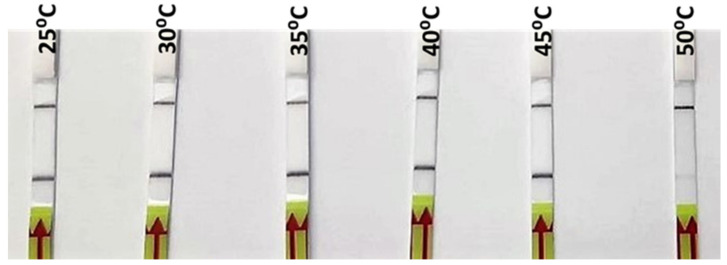
Lateral flow strips showing RPA-LFA results for the different incubation temperatures tested (25, 30, 35, 40, 45, and 50 °C).

DNA extracted from one of the reference samples of *O. vulgaris* was used for the optimization of the RPA incubation temperature. Six different incubation temperatures, from 25 °C to 50 °C, were tested. As can be seen in Figure 3, a strong signal was observed from 25 °C up to 45 °C, while at 50 °C, only a weak signal was produced (Figure 3). The selection of the incubation temperature, among those that gave a positive signal, was based on the recommendation by the manufacturer (40 °C), which gave a strong positive signal in the incubation test; furthermore, it was likely to be more specific than the lower ones. Subsequently, the rest of parameters (primers, probe and Mg concentrations) were adjusted for that temperature in order to obtain the highest specificity.

### 3.3. Detection Limit

**Figure 4:**
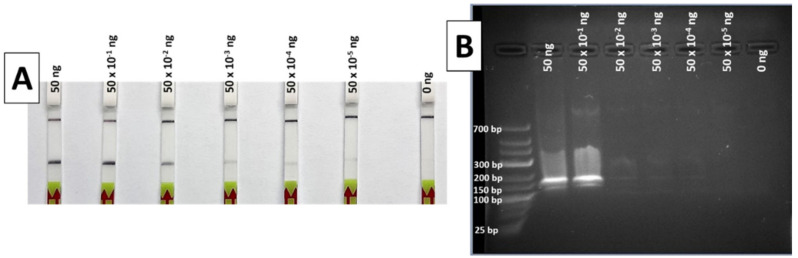
Results of the limit of detection test of the RPA method, with LFA (**A**) and 2% agarose gel (**B**). Total ng of DNA per reaction was 50, 50 × 10^−1^, 50 × 10^−2^, 50 × 10^−3^, 50 × 10^−4^, 50 × 10^−5^ and 0 ng, from left to right in both systems.

During the limit of detection (LOD) test, the results of the RPA-LFA show a gradually decreasing signal in the strip as the DNA concentration reduces. The lowest DNA template quantity that shows a clear and easily detectable positive band is 50 × 10^−2^ ng (Figure 4A), a quantity that can be considered the LOD of the method for the conditions used, although weak bands are also visible with 50 × 10^−3^ down to 50 × 10^−5^ ng of DNA.

When the same RPA products were visualized in a 2% agarose gel after electrophoresis, only the highest DNA quantities (50 and 5 ng) were easily detectable (Figure 4B). These results indicate a greater sensitivity for the LF detection compared to agarose gel electrophoresis. 

### 3.4. Specificity and Sensitivity

**Figure 5:**
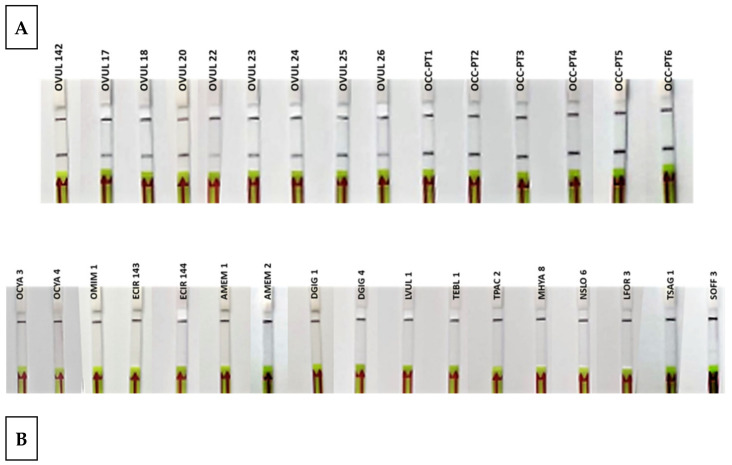
Lateral flow strips showing the RPA-LFA test results of reference samples (**A**) *Octopus vulgaris* samples (*n* = 15). (**B**) Related species and possible substitute species (*n* = 17). Control band (up) and test band (down) indicate that the assay is valid and that *Octopus vulgaris* is present in the sample.

During the test with reference samples, all *O. vulgaris* samples gave positive results, while all non-*O. vulgaris* samples were negative (Table 1 and Figure 5). These results represent the highest specificity and specificity levels, 100%, in both parameters, achieving the objective of successfully discriminating between all the species tested.

### 3.5. Application to Commercial Products

**Figure 6:**
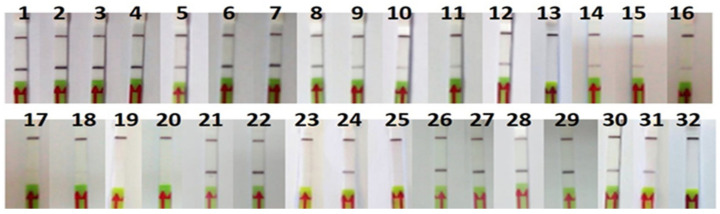
Lateral flow strips showing RPA-LFA test results of commercial samples (*n* = 32).

The RPA-LFA analysis of the commercial products tested were consistent with the FINS authentication (Table 2 and Figure 6). One of the canned samples (P11) could not be authenticated by sequencing because of a PCR failure, but the result with the RPA-LFA method was positive for *O. vulgaris* and consistent with the label. Regarding mislabeling, one of the frozen samples labeled as *O. vulgaris* (P32) was authenticated by FINS as *Octopus maya*, also showing a negative result with the RPA-LFA method, consistent with the FINS result but not with the label, indicating a case of species substitution. Another type of mislabeling was also found in one sample (P23) of *Eledone cirrhosa* collected in Spain; in this case, the label had the wrong commercial name “pulpito” (meaning “little octopus”), which is not accepted by the Spanish legislation [29]. It was also noted that all non-processed samples (frozen and defrosted) showed the scientific name on the label, while 57% of the processed samples (cooked and canned) did not, although it is not legally required in the latter.

Although the initial template DNA quantity was the same in all RPA reactions, some differences in the intensity of the signal were observed, usually weaker in processed products (Figure 6).

### 3.6. Interlaboratory Validation

During the interlaboratory validation test with blind samples, different DNA extraction methods and DNA quantification procedures were used by the different laboratories. The results from all laboratories were considered valid, since positive and negative controls were correct. The intensity of the positive bands differed among laboratories; however, as described in the Materials and Methods section, only clear and distinguishable bands were considered positive. The results show five cases of false positives (in red) and none of the false negatives were found, giving the method a specificity of 90% and a sensitivity of 100% in the laboratories (Table 4). Two species in particular (*Dosidicus gigas* and *Eledone cirrhosa*) caused the false positives (see Supplementary Materials for more information about the interlaboratory validation results). 

## 4. Discussion

The results obtained by the authors in the internal tests show 100% specificity and 100% sensitivity (Table 1; Table 2), while the interlaboratory trial also indicated 100% sensitivity, but a lower specificity (90%) (Table 4). This decrease in specificity could be due to the low levels of contamination of *O. vulgaris* template DNA or during the RPA amplification, whereby false-positives can occur as a result of primer dimers that carry the biotin [30]. The fact that in some laboratories, all samples showed the correct results while the samples in others did not indicates that the differences might also lie in small procedure deviations and differences in DNA quality. For example, some laboratories used DNA quantification methods which tend to overestimate the concentration, such as absorbance at 260 nm, which implies that the real quantity used is lower than the recommended quantity, and they still had successful results. These results add robustness to the method, since it can be implemented with different quantification methodologies and different DNA quantities. Nevertheless, it is important to note that it is not a quantitative method, since band intensity is also affected by other factors, such as type of processing or sample degradation, which is why all analyses need to be accompanied by positive and negative controls to be used as reference, and DNA quantity must be equal in all samples. Additionally, although the LOD has been stablished as 0.5 ng (Figure 4), the recommended quantity for detection is 50 ng, since the rest of parameters have been optimized for that quantity. In terms of specificity, the high performance of the designed RPA system allows many optimization possibilities (DNA template quantity, amplification temperature and time, oligos and reagents concentrations, etc.), which suggest that specificity can be increased for particular laboratories or control points by making small adjustments for a more stringent amplification. Some authors have also found that the addition of betaine to the reaction can correct false positives but may influence assay sensitivity [31]. Special attention should be paid to the species *Eledone cirrhosa* and *Dosidicus gigas* for optimization, since these species seem to be more susceptible to causing false positives (Table 4), and this must be taken into account during the optimization and implementation in a particular laboratory or at a control point. However, the total absence of false negatives presents a great advantage in terms of official controls, since there is no risk of charging an honest trader unfairly.

The most common substitutes for *O. vulgaris*, as seen in the literature and authors’ experience, are *Dosidicus gigas* [15] and *Amphioctopus* spp. [11]. It is particularly well known that the arms of *D. gigas*, chopped and cooked, have an octopus-like appearance, and given that this squid is the most captured cephalopod in the world and the price is much lower than the price of the common octopus, this is a clear example of economic fraud. The presented method has proved to be efficient in differentiating both mentioned species, which implies a major step in the fight of this type of seafood fraud. Having said that, this study has tested the species considered of importance in the European markets; however, in order to implement this tool at control points of other countries, other problematic species should be included in the optimization of the specificity. The method can detect very low DNA quantities, which can be very useful in the field to be used with less efficient DNA extraction methods or degraded samples. The detection limit assay also shows the potential of the technique for semiquantitative determination, with the intensity of the test band being an indication of the initial DNA quantity. However, this should be further investigated to take into account the presence of PCR inhibitors or the type of product, since DNA degradation due to the processing also affects the band intensity, as seen in the application of the method to commercial products in this work.

The tests at different temperatures show a successful amplification in a wide range, including room temperature (25 °C) and body temperature (35–40 °C) (Figure 3). The implications of these results include the possibility of optimization at different conditions with very simple equipment or without any equipment at all, making it possible to run the complete analysis at room temperature, which is particularly important for field control points or laboratories with limited resources.

The rapidity of the presented protocol stands out as one of the main advantages, taking less than 40 min from purified DNA to result. Depending on the DNA extraction method chosen, hundreds of samples can be analyzed in one working morning.

The tests carried out with products obtained at the points of sale also shows that the designed system works for all types of products, including those that have undergone a high temperature processing (e.g., canning) and have failed in the amplification by conventional PCR. Therefore, this is a good alternative for the authentication of canned *O. vulgaris* products.

Regarding labels, the samples analyzed mostly comply with European legislation in terms of the pieces of information that must be given to the consumers [32], since all products sampled had a declaration of the species, either with the scientific name (mandatory in fresh, frozen and chilled products) and/or the commercial name (Table 2). In Europe, the inclusion of the scientific name on the labels of cans is not mandatory, and the commercial names allowed in Spain (where the market sampling took place) in the case of cephalopods are not specific. In the particular case of canned octopus, several species can be traded under the umbrella name “Pulpo” [33], which can mislead consumers. Nevertheless, Spanish authorities have disclosed a plan to update this national regulation, including a new list of accepted commercial names for canned seafood [34], where the commercial name “Pulpo” will only allow the species *O. vulgaris*, making rapid authentication methods for this species even more relevant to monitor the compliance of this type of products with this new piece of legislation.

In the market sampling for this study, only one case of species substitution was found, a frozen sample labeled as *O. vulgaris* which was authenticated as *O. maya*. The calculated mislabeling rate is, therefore, 3%. This rate is lower than the rates found in previous studies [6,10], but we cannot assess that there has been a labeling improvement, as these results are not comparable with the present work, since the previous studies were conducted with a different sampling design. More studies that focus on each type of octopus product in different countries are needed to clarify the actual mislabeling rate on the market.

## 5. Conclusions

This work presents the development of a rapid, portable and easy-to-use method to detect the presence of *O. vulgaris* in food products.

The method has proven to be specific for *O. vulgaris* when compared with the species tested in this work, which include the main reported substitutes and related species. Nevertheless, the cephalopod market is global and hundreds of species are commercialized; therefore, other probable substitute species should be tested for markets outside of Europe. The method works on a wide range of incubation temperatures with different types of food processing, and can be performed by personnel with limited training using basic laboratory equipment.

The quantitation potential of RPA-LFA should be further explored, considering not only the presence of PCR inhibitors for different processing methods or for the presence of PCR inhibitors, but also of the DNA degradation after using different processing methods, since the results obtained in this work show differences in band intensity which can not only be attributable to DNA quantity.

The method has been validated internally and with an interlaboratory test in eight laboratories across five countries, presenting high sensitivity and specificity. Nevertheless, the results of this work highlight the importance of optimization for particular laboratories and the need for staff to be scrupulous with the protocol.

## Figures and Tables

**Table 1:** List of the reference samples used in this study. The table shows the corresponding species authenticated by FINS, the GenBank Accession number for the COI sequence and the RPA-LFA test result of each sample (*n* = 32).

Sample Code	Species (Authenticated by FINS)	GenBank Accession Number	RPA-LFA Result
**OVUL142**	*Octopus vulgaris*	MN977138	+
**OVUL 17**	*Octopus vulgaris*	MN977136	+
**OVUL 18**	*Octopus vulgaris*	MN977137	+
**OVUL 20**	*Octopus vulgaris*	MN977139	+
**OVUL 22**	*Octopus vulgaris*	MN977140	+
**OVUL 23**	*Octopus vulgaris*	MT919755	+
**OVUL 24**	*Octopus vulgaris*	MT919756	+
**OVUL 25**	*Octopus vulgaris*	MT919757	+
**OVUL 26**	*Octopus vulgaris*	MT919758	+
**OCC-PT1**	*Octopus vulgaris*	MT919759	+
**OCC-PT2**	*Octopus vulgaris*	MT919760	+
**OCC-PT3**	*Octopus vulgaris*	MT919761	+
**OCC-PT4**	*Octopus vulgaris*	MT919762	+
**OCC-PT5**	*Octopus vulgaris*	MT919763	+
**OCC-PT6**	*Octopus vulgaris*	MT919764	+
**OCYA 3**	*Octopus cyanea*	MN977143	-
**OCYA 4**	*Octopus cyanea*	MN977144	-
**OMIM1**	*Octopus mimus*	MN977146	-
**ECIR 143**	*Eledone cirrhosa*	MN977149	-
**ECIR 144**	*Eledone cirrhosa*	MN977150	-
**AMEM 1**	*Amphioctopus membranaceus*	MN977147	-
**AMEM 2**	*Amphioctopus membranaceus*	MT919765	-
**DGIG 1**	*Dosidicus gigas*	MN977152	-
**DGIG 4**	*Dosidicus gigas*	MN977153	-
**LVUL 1**	*Loligo vulgaris*	MN977128	-
**TEBL 1**	*Todaropsis eblanae*	MN977179	-
**TPAC 2**	*Todarodes pacificus*	MT919767	-
**MHYA 8**	*Martialia hyadesii*	MN977155	-
**NSLO6**	*Nototodarus sloanii*	MN977156	-
**LFOR 3**	*Loligo forbesii*	MT919766	-
**TSAG 1**	*Todarodes sagittatus*	MN977180	-
**SOFF 3**	*Sepia officinalis*	MN977162	-

A positive symbol (+) indicates that *Octopus vulgaris* was detected by the RPA-LFA test, and a negative symbol (-) indicates that a non-*O. vulgaris* species was found.

**Table 2:** Analyzed commercial samples with the indication of the type of sample and type of retailers where they were obtained (S: supermarket, R: restaurant), commercial name of the product (translated into English) and scientific name (if present, NI (Not indicated) if absent). The table also shows the FINS authentication results together with the GenBank accession number and RPA-LFA results.

Sample Code	Type of Sample	Retailer	Commercial Name (on Label)	Scientific Name on Label)	FINS Authentication	Accession Number GenBank	RPA-LFA Result
**P1**	Cooked	S	Cooked octopus	*Octopus vulgaris*	*Octopus vulgaris*	MT919738	+
**P2**	Frozen	S	Raw octopus	*Octopus vulgaris*	*Octopus vulgaris*	MT919739	+
**P3**	Cooked	S	Cooked octopus legs	NI	*Octopus vulgaris*	MT919740	+
**P4**	Cooked	S	Cooked octopus	*Octopus vulgaris*	*Octopus vulgaris*	MT919741	+
**P5**	Canned	S	Octopus in olive oil	*Octopus vulgaris*	*Octopus vulgaris*	*	+
**P6**	Canned	S	Octopus in olive oil from Galician estuaries	NI	*Octopus vulgaris*	*	+
**P7**	Cooked	R	Octopus “á feira”	NI	*Octopus vulgaris*	MT919742	+
**P8**	Cooked	S	Octopus legs cooked in their juice	*Octopus vulgaris*	*Octopus vulgaris*	MT919743	+
**P9**	Cooked	S	Cooked chopped octopus, Galician style	NI	*Octopus vulgaris*	MT919744	+
**P10**	Canned	S	Octopus “Galician style”	NI	*Octopus vulgaris*	*	+
**P11**	Canned	S	Octopus in garlic	NI	amplification failure		+
**P12**	Canned	S	Octopus in extra virgin olive oil	*Octopus vulgaris*	*Octopus vulgaris*	*	+
**P13**	Canned	S	Octopus from the Cantabrian Sea	NI	*Octopus vulgaris*	*	+
**P14**	Cooked	R	Octopus “á feira”	NI	*Octopus vulgaris*	MT919745	+
**P15**	Cooked	R	Cooked octopus	NI	*Octopus vulgaris*	MT919746	+
**P16**	Canned	S	Octopus in olive oil	*Octopus vulgaris*	*Octopus vulgaris*	*	+
**P17**	Canned	S	Squid cubes in seafood sauce	*Dosidicus spp*	*Dosidicus gigas*	*	-
**P18**	Canned	S	Cubes in garlic octopus style	*Dosidicus gigas*	*Dosidicus gigas*	*	-
**P19**	Canned	S	Pieces of Jumbo flying squid tentacles in Galician sauce	*Dosidicus gigas*	*Dosidicus gigas*	*	-
**P20**	Canned	S	Cubes in sunflower oil. Jumbo flying squid.	NI	*Dosidicus gigas*	*	-
**P21**	Cooked	S	Sliced Wild Octopus	*Octopus vulgaris*	*Octopus vulgaris*	MT919747	+
**P22**	Defrosted	S	Thawed octopus	*Octopus vulgaris*	*Octopus vulgaris*	MT919748	+
**P23**	Fresh	S	Fresh “pulpito”	*Eledone cirrhosa*	*Eledone cirrhosa*	MT919749	-
**P24**	Canned	S	Octopus in olive oil	NI	*Octopus vulgaris*	*	+
**P25**	Canned	S	Squid cubes in garlic	*Dosidicus spp*	*Dosidicus gigas*	*	-
**P26**	Cooked	S	Cooked chopped octopus	NI	*Octopus vulgaris*	MT919750	+
**P27**	Canned	S	Octopus in olive oil	NI	*Octopus vulgaris*	*	+
**P28**	Canned	S	Octopus in seafood sauce	NI	*Octopus vulgaris*	*	+
**P29**	Frozen	S	Frozen Octopus	*Octopus vulgaris*	*Octopus vulgaris*	MT919751	+
**P30**	Frozen	S	Frozen Octopus	*Octopus vulgaris*	*Octopus vulgaris*	MT919752	+
**P31**	Grilled	R	Grilled octopus	NI	*Octopus vulgaris*	MT919753	+
**P32**	Frozen	S	Ultrafrozen Raw octopus	*Octopus vulgaris*	*Octopus maya*	MT919754	-

A positive symbol (+) indicates that *O. vulgaris* was detected by the RPA-LFA test, and a negative symbol (-) indicates that a non-*Octopus vulgaris* species was found. Words in red font indicate mislabeling. * Canned samples were sequenced with the 16S fragment for FINS authentication and are not available in Genbank because it does not allow short length sequences.

**Table 3:** Primers used for FINS authentication of all the samples.

Sequences	Region	Reference
LCO1490-5′GGTCAACAAATCATAAAGATATTGG3′ HCO2198-5′TAAACTTCAGGGTGACCAAAAAATCA3′	Mitochondrial COI	Folmer, 1994 [24]
16SVAR-F- 5′CAAATTACGCTGTTATCCCTATGG3′ 16SVAR-R- 5′GACGAGAAGACCCTAATGAGCTTT3′	Mitochondrial 16S rDNA	Chapela et al., 2002 [12]

**Table 4:** Interlaboratory validation results. The table shows the species in the samples and the result achieved by the RPA-LFA test. Control positive (C+) and control negative (C--), to check the specificity of the reaction, were also included.

Sample Code	Species	LAB 1	LAB 2	LAB 3	LAB 4	LAB 5	LAB 6	LAB 7	LAB 8
IIM1	*Amphioctopus membranaceus*	-	-	-	-	-	-	-	-
IIM2	*Dosidicus gigas*	-	-	-	+	-	-	+	-
IIM3	*Octopus vulgaris*	+	+	+	+	+	+	+	+
IIM4	*Octopus mimus*	-	-	-	-	-	-	-	-
IIM5	*Octopus vulgaris*	+	+	+	+	+	+	+	+
IIM6	*Octopus vulgaris*	+	+	+	+	+	+	+	+
IIM7	*Octopus cyanea*	-	-	-	-	-	-	-	-
IIM8	*Eledone cirrhosa*	+	-	-	-	+	-	+	-
C+	*Octopus vulgaris*	+	+	+	+	+	+	+	+
C-	*Nototodarus sloanii*	-	-	-	-	-	-	-	-

A positive symbol (+) indicates that *O. vulgaris* was detected, and a negative symbol (-) indicates that a non-*Octopus vulgaris* species was found by the eight participant laboratories. Red symbols show the false positives obtained.

## Supplementary Materials

The following are available online at https://www.mdpi.com/article/10.3390/foods10081825/s1, Document 1: SOP for the detection/authentication of *Octopus vulgaris* in foodstuffs by Recombinase Polymerase Amplification (RPA) and Lateral Flow assay (LFA).
