# Supplementary material for: A New Rapid Method for the Authentication of Common Octopus (Octopus vulgaris) in Seafood Products Using Recombinase Polymerase Amplification (RPA) and Lateral Flow Assay (LFA)
Source: Foods. 2021 Aug 6;10(8):1825. doi: 10.3390/foods10081825 (PMC8394702; doi:10.3390/foods10081825)
Supplement: Supplementary file 1 [file foods-10-01825-s001.zip › foods-1285084-supplementary.pdf]

Standard Operating Procedure for the detection/authentication of *Octopus vulgaris* in foodstuffs by Recombinase Polymerase Amplification (RPA) and Lateral Flow assay (LFA)

Amaya Velasco[1], Graciela Ramilo-Fernández[1], Françoise Denis[2], Luis Oliveira[3], Peter Shum[4], Helena Silva[3] Oscar Blanco[5], Stefano Mariani[4], Miguel Angel Pardo[6], Regina Klapper[7], Ute Schröder[7], Julien Landure[8], and Carmen G. Sotelo[1]

1 Instituto de Investigaciones Marinas (IIM-CSIC), Eduardo Cabello 6, 36208 Vigo (Pontevedra), Spain; amayavelasco@iim.csic.es (A. V.); graciela@iim.csic.es (G.R.F.); carmen@iim.csic.es (C.G.S.)

2 BOREA MNHN, CNRS 8067, SU, IRD 207, UCN, UA- BIOSSE Le Mans Université; francoise.denis@univ-lemans.fr (F. D.)

3 Instituto Português do Mar e da Atmosfera (IPMA, I.P.), Av. Dr. Alfredo Magalhães Ramalho 6, 1495-165 Algés, Portugal; hsilva@ipma.pt (H. S.); luis.oliveira@ipma.pt (L. O.)

4 School of Biological and Environmental Sciences, Liverpool John Moores University, Liverpool, UK; p.shum@ljmu.ac.uk (P. S.); S.Mariani@ljmu.ac.uk (S. M.)

5 Centro de Investigación y Control de la Calidad (CICC), Ministerio de Sanidad, Consumo y Bienestar social, Avenida de Cantabria 52, 28042 Madrid, Spain; oblanco@mscbs.es (O. B.)

6 AZTI-Fisheries and Food Technological Institute, Txatxarramendi ugartea z/g, E-48395 Sukarrieta (Bizkaia), Spain; mpardo@azti.es (M. A. P.)

7 Max Rubner-Institut (MRI) Federal Research Institute of Nutrition and Food, Department of Safety and Quality of Milk and Fish Products, Hermann-Weigmann-Straße 1, 24103 Kiel, Germany; Regina.Klapper@mri.bund.de (R. K.); Ute.Schroeder@mri.bund.de (U. S.)

8 Service commun des laboratoires (DGCCRF – DGDDI) 146 Traverse Charles Susini, Marseille, France; Julien.landure@scl.finances.gouv.fr (J. L.)

SEATRACES: Project funded by the Interreg Atlantic Area Programme through the European Regional Development Fund. EAPA_87/2016

CONTENTS

Background

Scope

Abbreviations

Principle of the method

Materials and equipment

Procedures

Validation

References

End notes

BACKGROUND

SEATRACES is an EU Interreg Atlantic Area project with the aim of demonstrating to stakeholders and consumers that Labeling and Traceability are essential to protect and valorize Atlantic Area's fisheries and aquaculture. Illegal fisheries, fraud and mislabeling are the main issues representing a serious risk to the existence of this important economic activity for the Atlantic Area Regions, thus, one of SEATRACES' purposes is the prevention of fraud by developing new, validated and standardized authentication techniques for the control of seafood labeling.

SCOPE

This SOP describes a method for the authentication and detection of *Octopus vulgaris* DNA in foodstuffs with the purpose to provide a rapid and effective tool for food authenticity control. It has been tested in 15 species (see list below) and works for fresh, frozen, cooked and grilled products, as well as for highly processed samples such as canned. The method is capable to distinguish between very closely related species within the *Octopus* genus, therefore, the probability of obtaining false positives with non-tested species is expected to be low.

List of species tested:

**Scientific name**	**Common name (FAO)**
Octopus vulgaris	Common octopus
Octopus cyanea	Big blue octopus
Octopus mimus	Changos octopus
Octopus maya	Mexican four-eyed octopus
Amphioctopus membranaceus	Webfoot octopus
Eledone cirrhosa	Horned octopus
Dosidicus gigas	Jumbo flying squid
Loligo vulgaris	European squid
Loligo forbesii	Veined squid
Todaropsis eblanae	Lesser flying squid
Todarodes pacificus	Japanese flying squid
Todarodes sagittatus	European flying squid
Martialia hyadesi	Sevenstar flying squid
Nototodarus sloanii	Wellington flying squid
Sepia officinalis	Common cuttlefish

ABBREVIATIONS

SOP: Standard Operating Procedure

DNA: Deoxyribonucleic acid

RPA: Recombinase Polymerase Amplification

PCR: Polymerase Chain Reaction

COI: Mitochondrial Cytochrome c Oxidase 1 gene

LFA: Lateral Flow assay

SSB: single-stranded DNA-binding protein

FAM: carboxyfluorescein, single isomer

FICT: fluorescein-5,6-isothiocyanate, mixed isomers

PRINCIPLE OF THE METHOD

RPA (Recombinase Polymerase Amplification) is a rapid and isothermal alternative to conventional PCR which achieves the amplification by using three types of proteins: a recombinase, a single-stranded DNA-binding protein (SSB) and a polymerase. The recombinase (usually *Escherichia coli* recA) can bind to primer oligonucleotides and stimulate the resulting recombinase-primer complex to search for homologous sequences in duplex DNA. Once homology is located, SSB proteins bind to the displaced strand, and the oligonucleotide is paired to its complement permitting the strand-displacing polymerase to begin synthesis from the 3’ end. This process is cyclic and allows an exponential amplification process (Piepenburg et al, 2006).

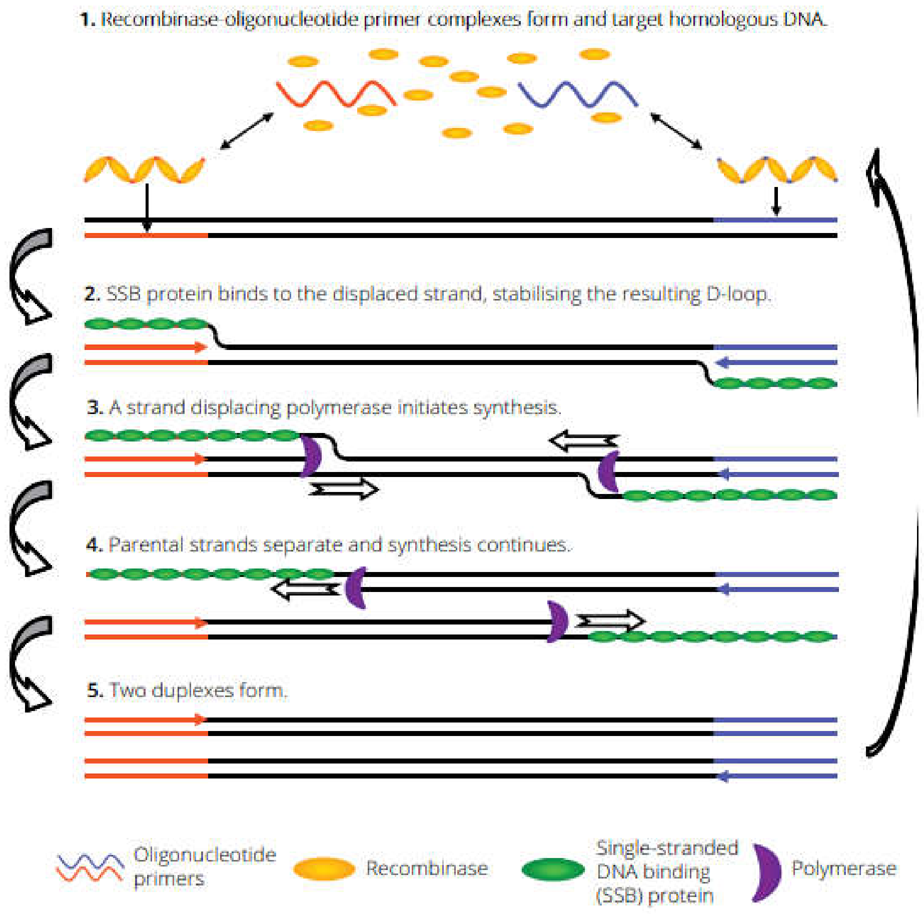

Figure S1: RPA cycle. Source: TwistAmp® DNA Amplification Kits Combined Instruction Manual

For the endpoint detection of amplification, traditional methods such as electrophoresis can be used, but some commercial RPA kits have been designed for a detection by sandwich assays, such as lateral flow technology-based systems, which require special and compatible probes but has some advantages such as higher sensitivity and simplicity. The probe is an additional oligonucleotide which is typically homologous to sequences between the main amplification primers and can therefore bind to the amplification product. The probe is typically labelled with FAM or FITC, and the reverse primer is labelled with Biotin or DIG, thus the resulting amplicon carries a double label which can be detected in a LFA (Lateral Flow assay).

The following figure shows the functioning of the LFA for the detection of these modified amplicons.

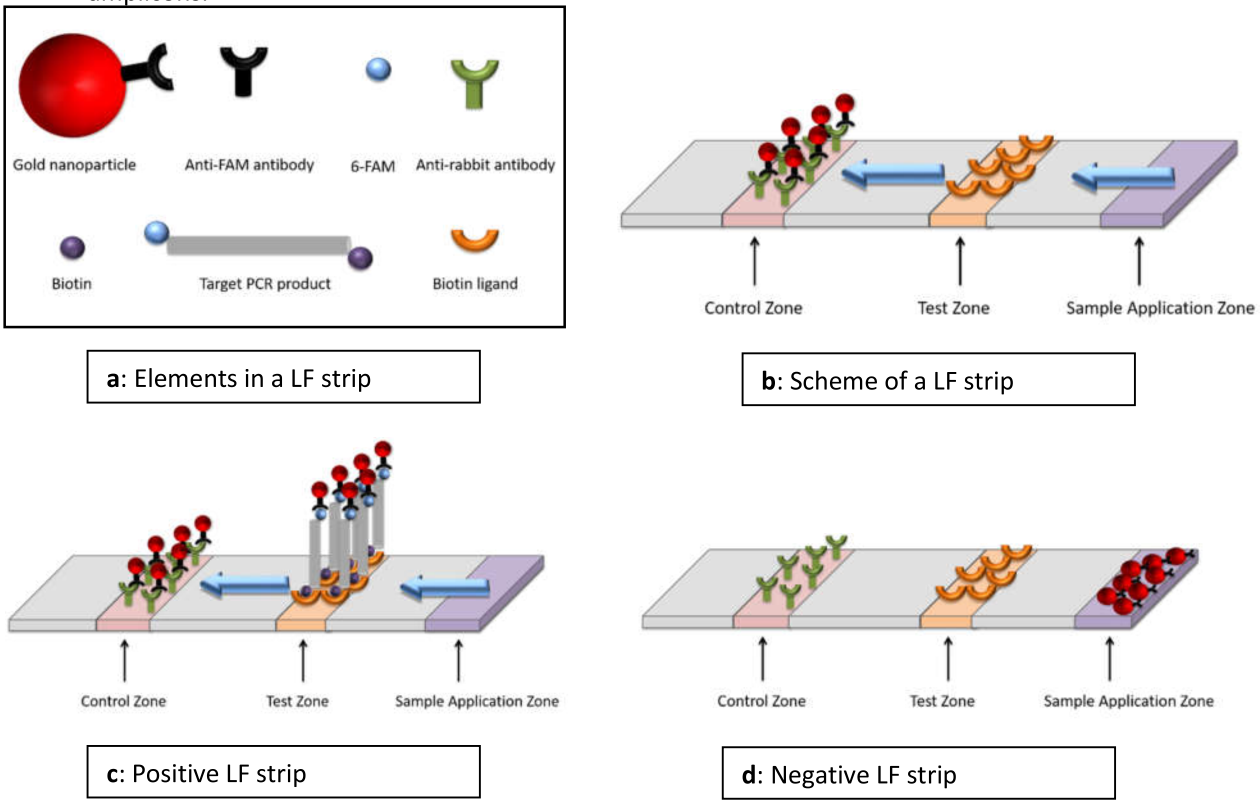

Figure S2: LFA principle. A gold particle with the attached anti-FAM antibody binds to the labeled amplicon. By capillarity, the gold complex diffuses over the membrane and reaches the test band with the biotin-ligand. Only the gold complexes with labelled amplicons will be immobilized by biotin-ligand molecules at the test band and will therefore generate a band over the time. Not-captured gold particles flow over the control band and will be fixed there by the antibodies. Source: Taboada et al. 2017. *Food Chem*. 233: 182-189.

MATERIALS AND EQUIPMENT

Reagents and solutions

- Molecular grade water
- Lysis buffer (1% SDS, 150 mM NaCl, 2 mM EDTA, and 10 mM Tris-HCl at pH 8)
- Guanidinium isothiocyanate 5M
- Proteinase K (20 mg/ml)
- 80% isopropanol
- Amplification primers and probe
  The system designed for the detection of Octopus vulgaris amplifies a 155 bp fragment of the COI region.
  Primers and probe sequences and modifications:
  OVUL_F1_nfo: 5'-ACTAGGAGCACCAGATATAGCATTCCCACGAATA-3'
  OVUL_R1_nfo: Biotin-5'-GAGCTAAATTTCTTGAAAGAGGCGGGTAAACGGT-3'
  OVUL_ P1_nfo: 5'-ACTCTTACCTCCTTCTCTTACTCTTCTCCTTT[THF]ATCTGCAGCAGTTGA-3'-FAM

  The reverse primer is modified with a Biotin in 3'.
  The probe has a 6-FAM modification in 3' and a blocking group in 3' (Spacer C3). THF correspond to the position of the tetrahydrofuran residue, which is the substrate for the enzime.
  Primers and probe can be ordered from various oligonucleotide manufacturers with the order forms in this link:
  https://www.twistdx.co.uk/en/support/rpa-assay-design-2/twistamp-basic-oligo-forms/twistamp-nfo-oligo-forms
- *Octopus vulgaris* DNA for positive control

Commercial kits

- Wizard DNA Clean up system (Promega)
- Qubit dsDNA BR assay kit (ThermoFisher)
- TwistAmp nfo kit (TwisDX)
- Milenia HybriDetect - Universal Lateral Flow Assay Kit (Milenia Biotec)

Disposable material

- Pipette tips (containing protective filters for PCR)
- 1,5 ml tubes
- Qubit assay tubes (ThermoFisher)
- Reaction tubes or 96-well microtiter plate

Other materials

- Scalpel
- Tweezers

Equipment

- Precision pipettes

- Vortex shaker
- Thermal shaker
- Thermal cycler
- Microcentrifuge
- Vacuum manifold
- Qubit fluorometer (ThermoFisher) or other equivalent equipment for DNA quantification

PROCEDURES

- **Sample preparation**
  Tissue must be separated from other ingredients and any sauce or oil should be removed by immersion in molecular grade water. This washing step should be repeated if needed until obtaining a clean muscular tissue.

- **DNA extraction**
  The method has been tested using Wizard DNA Clean up system (Promega), following the manufacturer's instructions. The suitability of other extraction methods must be proved before its use.

  Procedure for the digestion of the tissue:
  1. Mince 0.3 g of muscle tissue with a scalpel and tweezers, and transfer to a 1,5ml tube
  2. Add 860 µl of lysis buffer (1% SDS, 150 mM NaCl, 2 mM EDTA, and 10 mM Tris-HCl at pH 8)
  3. Add 100 µl of guanidinium isothiocyanate 5 M
  4. Add 40 µl of proteinase K (20 mg/ml) and vortex to mix
  5. Incubate at 56 °C in a thermo shaker while shaking at 1000 rpm
  6. After 3 h, 40 µl of extra proteinase K was added and left overnight.

  Procedure for the DNA isolation:

  1. Centrifuge the digested tissue at 13000 rpm for 5 min
  2. Collect the supernatant in a sterile 1.5 ml microcentrifuge tube
  3. Centrifuge at 13000 rpm for 5 min
  4. Thoroughly mix the Wizard DNA resin and add 1 ml of resin to a 1.5 ml microcentrifuge tube
  5. Add 500 µl of the sample to the tube and mix by pipetting
  6. Use one Wizard minicolumn for each sample. Attach the provided syringe barrel to the extension of each minicolumn. Insert the tip of the minicolumn/syringe Barrel assembly into the vacuum manifold.
  7. Pipet the resin/sample mix into the Syringe Barrel. Apply a vacuum to draw the solution through the minicolumn.
  8. Add 2 ml of 80 % isopropanol to the syringe barrel, and re-apply a vacuum to draw the solution through the minicolumn.
  9. Dry the resin by continuing to draw a vacuum for 30 seconds after the solution has been pulled through the column. Remove the Syringe Barrel and transfer the minicolumn to a 1.5 ml microcentrifuge tube.

10. Centrifuge the minicolumn at 12,000 rpm in a microcentrifuge for 2 minutes to remove any residual isopropanol.
11. Transfer the minicolumn to a new microcentrifuge tube. Apply 50 µl of prewarmed (70 °C) water to the minicolumn and wait 1 minute.
12. Centrifuge the minicolumn for 20 seconds at 14,000 rpm to elute the DNA.
13. Remove and discard the minicolumn. The purified DNA may be stored at 4 °C or –20 °C.

- **DNA quantification**

  Although the system has proved to detect quantities from $50x10^{-4}$ ng of DNA, this SOP is designed for a total DNA template amount of 50 ng per sample, and the use of lower concentrations could entail weaker bands or false negatives. DNA concentrations should be quantified with Qubit fluorometer with the Qubit dsDNA BR assay kit; any other method for DNA quantification does not guarantee the reproducibility of the results.

  Procedure for DNA quantification:

  1. Set up the required number of 0.5-mL Qubit assay tubes (Cat. no. Q32856) tubes for standards and samples. The Qubit dsDNA BR Assay requires 2 standards. Label the lid of each standard tube and sample tubes.
  2. Prepare the Qubit working solution by diluting the Qubit dsDNA BR Reagent 1:200 in Qubit dsDNA BR Buffer.
     NOTE: The final volume in each tube must be 200 µL. Each standard tube requires 190 µL of Qubit working solution, and each sample tube requires anywhere from 180–199 µL. Prepare sufficient Qubit working solution to accommodate all standards and samples. For example, for 8 samples, prepare enough working solution for the samples and 2 standards: 200 µL per tube in 10 tubes yields 2 mL of working solution (10 µL of Qubit reagent plus 1990 µL of Qubit buffer).
  3. Add 190 µL of Qubit working solution to each of the tubes used for standards.
  4. Add 10 µL of each Qubit standard to the appropriate tube, then mix by vortexing 2–3 seconds. Be careful not to create bubbles.
  5. Add 198 µL of Qubit working solution to individual assay tubes.
  6. Add 2 µL of each sample to the appropriate tube so that the final volume in each tube after adding sample is 200 µL, then mix by vortexing 2–3 seconds.
  7. Allow all tubes to incubate at room temperature for 2 minutes
  8. On the Home screen of the Qubit Fluorometer, press DNA, then select dsDNA and Broad Range as the assay type.
  9. Measure Standard 1 and Standard 2.
  10. Measure the samples, selecting 2 µL as sample volume.

- **RPA**

  A positive control with DNA from a reference sample of *Octopus vulgaris* and a sample of a non-target species must be run with the set of samples. For the monitoring of possible contaminations, a negative control is also necessary for each RPA mix.
  Dilute DNA samples to a concentration of 50 ng/µl.

1. Prepare the following RPA reaction mix as follows taking into account that is for just one sample reaction, therefore it will require adapting it if another number of samples is being analysed. It is also recommended to add 10% to the total volume of each of the reagents utilised, in order to account for pipetting error):

Forward primer (10 µM)	1.8 µl
Reverse primer (10 µM)	1.8 µl
nfo probe (10 µM)	0.6 µl
Primer Free Rehydration buffer	29.5 µl
water	13.1 µl
**Total volume**	**46.8 µl**

Vortex and spin briefly.

2. For each sample, transfer 46.8 µl of this mix to the tube with the reaction pellet. Mix by pipetting up and down until the entire pellet has been resuspended.

3. Add 1 µl of template DNA (50 ng/µl) to the tube.

4. For each sample, add 2.2 µl of 280 mM magnesium acetate (MgOAc) (provided with the kit). In the case of dealing with many samples, it is recommended to do this simultaneously by placing the MgOAc into the lid of the reaction tubes (strip of 8), then cap the tubes carefully. Vortex briefly and spin down.
   NOTE: Reactions are activated using MgOAc. The RPA reaction starts as soon as MgOAc is added, even at room temperature. Proceed quickly to incubation step once MgOAc has been added.

5. Incubate the tubes in a thermal cycler at 40 °C for 15 minutes.
   NOTE: The method has been validated in a thermal cycler, the use of other non-calibrated heater could lead to different results.

- **Lateral flow detection**

  NOTE: As a precaution to avoid contamination, perform all post amplification work in a separate area to the RPA reaction setup.

  1. Remove 1 µl of reaction and mix with 49 µl PBST running buffer (supplied in the Milenia HybriDetect Kit) in a microcentrifuge tube.
  2. Transfer 10 µl of the diluted sample to the sample pad end of the Hybridetect strip. The strips can be previously labeled in the labeling area, at the opposite end of the strip.

3. Place the strip vertically with the sample pad submerged in 150 µl of PBST running buffer. This can be done in open tubes, but in the case of many samples it is convenient to dispense the PBST into wells of a 96-well plate and stand the strips in the wells (see picture below).

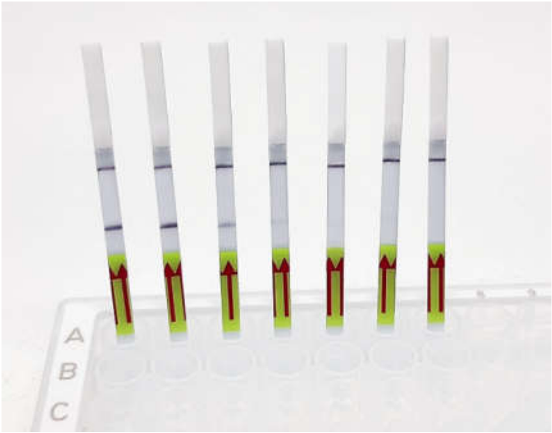

4. After 4 minutes at room temperature, transfer de strips to a white background for an immediate interpretation of the results. A photograph of the strips at this instant is highly recommended.

- **Interpretation of results**

  The presence of the amplification product is indicated by the development of a coloured test line if the template DNA belongs to *Octopus vulgaris*. A separate control line found further up the strip should always develop confirming that the strips are functioning correctly. If the test band is not clear or it is very faint in the established time, the result should be considered negative.

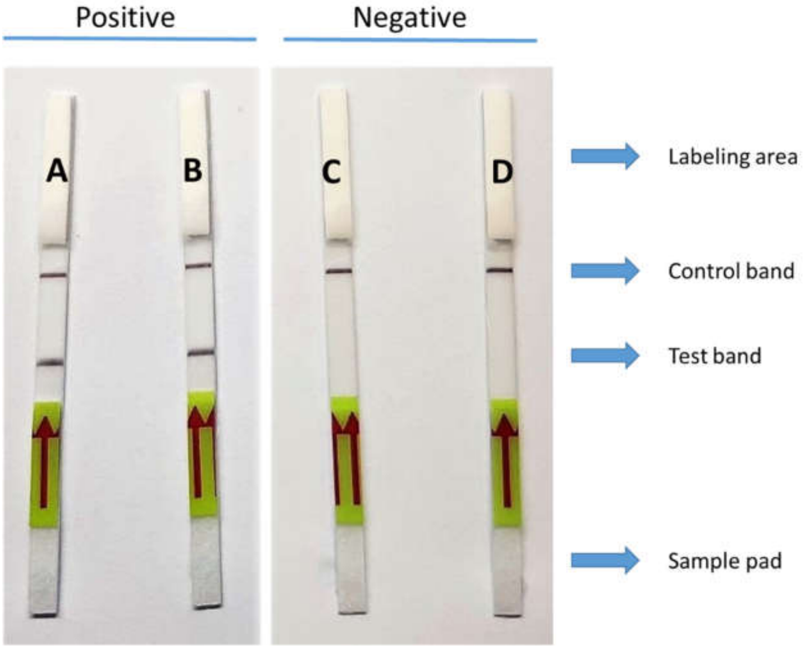

Figure: Interpretation of results. A: *Octopus vulgaris*; B: Positive control (*Octopus vulgaris*); C: *Dosidicus gigas*; D: Negative control (no DNA)

VALIDATION

This SOP has undergone an internal validation in 32 commercial samples from supermarkets and restaurants from Spain, which included different grades of processing: unprocessed (fresh), frozen, thawed, cooked and canned. All samples were also authenticated by FINS. Results obtained were all consistent with the FINS identification, and only samples containing *Octopus vulgaris* gave positive results.

It has also been tested in an interlaboratory study with 8 participant laboratories with 8 blind tissue samples and 2 control samples.

Participant laboratories:

Instituto de Investigaciones Marinas (IIM-CSIC), Spain
Max Rubner-Institut (MRI), Germany
Instituto Português do Mar e da Atmosfera (IPMA), Portugal
Centro de Investigación y Control de la Calidad (CICC), Spain
AZTI, Spain
Service commun des laboratoires (DGCCRF – DGDDI), France
Liverpool John Moores University (LJMU), United Kingdom
Muséum national d'Histoire naturelle (MNHN), France

Blind and control samples and results obtained:

SAMPLE CODE	SPECIES	LAB 1	LAB 2	LAB 3	LAB 4	LAB 5	LAB 6	LAB 7	LAB 8
IIM1	*Amphioctopus membranaceus*	NEGATIVE	NEGATIVE	NEGATIVE	NEGATIVE	NEGATIVE	NEGATIVE	NEGATIVE	NEGATIVE
IIM2	*Dosidicus gigas*	NEGATIVE	NEGATIVE	NEGATIVE	POSITIVE	NEGATIVE	NEGATIVE	POSITIVE	NEGATIVE
IIM3	*Octopus vulgaris*	POSITIVE	POSITIVE	POSITIVE	POSITIVE	POSITIVE	POSITIVE	POSITIVE	POSITIVE
IIM4	*Octopus mimus*	NEGATIVE	NEGATIVE	NEGATIVE	NEGATIVE	NEGATIVE	NEGATIVE	NEGATIVE	NEGATIVE
IIM5	*Octopus vulgaris*	POSITIVE	POSITIVE	POSITIVE	POSITIVE	POSITIVE	POSITIVE	POSITIVE	POSITIVE
IIM6	*Octopus vulgaris*	POSITIVE	POSITIVE	POSITIVE	POSITIVE	POSITIVE	POSITIVE	POSITIVE	POSITIVE
IIM7	*Octopus cyanea*	NEGATIVE	NEGATIVE	NEGATIVE	NEGATIVE	NEGATIVE	NEGATIVE	NEGATIVE	NEGATIVE
IIM8	*Eledone cirrhosa*	POSITIVE	NEGATIVE	NEGATIVE	NEGATIVE	POSITIVE	NEGATIVE	POSITIVE	NEGATIVE
C+	*Octopus vulgaris*	POSITIVE	POSITIVE	POSITIVE	POSITIVE	POSITIVE	POSITIVE	POSITIVE	POSITIVE
C-	*Nototodarus sloanii*	NEGATIVE	NEGATIVE	NEGATIVE	NEGATIVE	NEGATIVE	NEGATIVE	NEGATIVE	NEGATIVE

The results obtained in this ring test were used for calculating the sensitivity and specificity of the method:

Specificity= True Negatives / (True Negatives + False positives) x 100 = **90 %**

Sensitivity=True Positives / (True Positives + False negatives) x 100 = **100 %**

All samples containing *O. vulgaris* gave positive results (Sensitivity 100 %), whereas a small rate of false positives was found (in red font in the table), belonging to samples of *Dosidicus gigas* and *Eledone cirrhosa*. In light of these results, authors recommend that DNA template quantity and RPA conditions (time, temperature, MgOAc concentration, primers and probe concentrations) must be optimised in each laboratory to increase specificity, taking special attention to this species.

Although the methods used by the participant laboratories regarding DNA extraction and quantification were diverse, these deviations from the SOP did not seem to affect the results.

REFERENCES

- Piepenburg, O., Williams, C.H., Stemple, D.L., Armes, N.A. (2006) DNA detection using recombination proteins. PLOS Biology 4(7): e204. https://doi.org/10.1371/journal.pbio.0040204
- Taboada, L., Sánchez, A., Pérez-Martín, R.I., Sotelo, C.G. (2017) A new method for the rapid detection of Atlantic cod (*Gadus morhua*), Pacific cod (*Gadus macrocephalus*), Alaska pollock (*Gadus chalcogrammus*) and ling (*Molva molva*) using a lateral flow dipstick assay, Food Chemistry 233: 182-189.

END NOTES

TwistAmp®, Twista® , and TwistAmp® probe are registered trademarks of TwistDx™. Use of the RPA process and probe technologies are protected by US patents 7,270,981 B2, 7,399,590 B2, 7,435,561 B2, 7,485,428 B2 and foreign equivalents in addition to pending patents.

SDS information Safety Data Sheet (SDS) information for TwistDx™ products is provided on the TwistDx™ website at twistdx.co.uk. SDS documents are not included with product shipments.
